# Application of Seemingly Unrelated Regression in Medical Data with Intermittently Observed Time-Dependent Covariates

**DOI:** 10.1155/2012/821643

**Published:** 2012-12-18

**Authors:** Sareh Keshavarzi, Seyyed Mohammad Taghi Ayatollahi, Najaf Zare, Maryam Pakfetrat

**Affiliations:** ^1^Department of Epidemiology, School of Health & Nutrition, Shiraz University of Medical Sciences, Shiraz 7153675541, Iran; ^2^Department of Biostatistics, School of Medicine, Shiraz University of Medical Sciences, Shiraz 7134845794, Iran; ^3^Department of Internal Medicine, School of Medicine, Shiraz University of Medical Sciences, Shiraz 7134845794, Iran

## Abstract

*Background*. In many studies with longitudinal data, time-dependent covariates can only be measured intermittently (not at all observation times), and this presents difficulties for standard statistical analyses. This situation is common in medical studies, and methods that deal with this challenge would be useful. *Methods*. In this study, we performed the seemingly unrelated regression (SUR) based models, with respect to each observation time in longitudinal data with intermittently observed time-dependent covariates and further compared these models with mixed-effect regression models (MRMs) under three classic imputation procedures. Simulation studies were performed to compare the sample size properties of the estimated coefficients for different modeling choices. *Results*. In general, the proposed models in the presence of intermittently observed time-dependent covariates showed a good performance. However, when we considered only the observed values of the covariate without any imputations, the resulted biases were greater. The performances of the proposed SUR-based models in comparison with MRM using classic imputation methods were nearly similar with approximately equal amounts of bias and MSE. *Conclusion*. The simulation study suggests that the SUR-based models work as efficiently as MRM in the case of intermittently observed time-dependent covariates. Thus, it can be used as an alternative to MRM.

## 1. Introduction 

 Time-dependent covariates are common in longitudinal studies, and they may be missing thoroughly for some observations times, along with the outcome variable. This is a special kind of missing covariate which is called sporadic or intermittent missing (i.e., subjects with missing observations between observed time points) [1–5]. Sometimes, these intermittent missing observations may occur due to the design of the study, since some of the time-dependent covariates are costly or may not be necessary to be measured at all time points, especially in medical studies [6–9]. For example, in determining the prognosis of hospitalized patients, it is necessary to measure some blood profiles at different time points because the costs of measurements are high or frequent measurements may cause harm to the patients. Moreover, in the case of hemodialysis patients, during the dialysis session, some factors such as blood pressure or levels of magnesium may be measured at frequent intervals for each patient, but others such as blood urea nitrogen (BUN) may be measured only at the beginning and the end of the study.

Most of the existing standard statistical models typically require covariates to be completely observed. However, this assumption is not realistic in the presence of intermittently observed time-dependent covariates [10]. Lack of simultaneous measurement of time-dependent covariates leads to unobserved covariates and unbalances the design matrix. In such situations, the covariate is considered to be missing, but in actuality, it is simply not measured. 

 For analyzing incomplete and unbalanced longitudinal data with numerical response variable, there are several reasonable methods including mixed-effect regression model (MRMs). MRM has several desirable features that make it useful in longitudinal research such as including the subjects with missing covariate across time in the analysis. While MRM does allow incomplete data across time, data must be complete within the given time point (in terms of both the dependent variable and covariates) for that time point to be included in the analysis [11]. To handle this problem of MRM, several naive simple approaches have been developed including (1) complete case analysis (CC) or list-wise deletion, (2) using baseline observation carried forward (BOCF), and (3) using last observation carried forward (LOCF) [12–16]. There are some studies that show the application of these naive methods such as that conducted by Roy and Lin [10] about their resulting asymptotic bias while we choose them for dealing with missing covariates. However, little is known about their performance in spite of the fact that they are a commonly used ways of imputation for data with missing time-dependent covariates.

 In addition, most of classical analytic techniques for intermittent observation of time-dependent covariates have centred on models using an individual formulation of multivariate linear models. Examples are the random-effect model proposed by Jennrich and Schluchter [17] or the proposed models of Roy and Lin [10, 12]. However, alternate methods can be expressed with respect to each observation time. This formulation introduces several correlated models with some similar and different parameters simultaneously, as in seemingly unrelated regression (SUR) models of Zellner [18]. There are some proposals based on this method for unbalanced and incomplete longitudinal data like the proposed models of Park and Woolson [19]. Although the application of their model included time-dependent covariates, they simply assumed that if response variable *y* for *i*th subject at time *j* is observed, then the covariate vector of *i*th subject at time *j* is always observed. In their study, missing observations are assumed to be missing at random. However, this condition is not possible in presence of intermittent observation of time-dependent covariates.

 In the present study, we intend to view the SUR model in the context of longitudinal data and to examine its performance in presence of intermittently observed time-dependent covariates which is a common situation in medical studies. For this purpose, we considered the situation where the outcome variable was observed completely, but the time-dependent covariates could be only measured at some of the observation times for all cases. Moreover, we considered continuous outcome and time-dependent covariate. We first performed the SUR-based model for longitudinal data with time-dependent covariates measured at the same time points, and, further, the performance of this model as compared with MRM was evaluated. We next proposed some naive approaches based on SUR model for longitudinal data with intermittently observed time-dependent covariates. Furthermore, the capabilities of the proposed models were compared to MRM under classic approaches for missing covariate including complete case analysis, BOCF, and LOCF. 

## 2. Methods

### 2.1. Mixed-Effect Regression Model

 Suppose that there are *n* subjects and *t* time points for which data are collected. Let *y*_*ij*_ be a response variable and *X*_*ij*_ = (*x*_*ij*1_, ..., *x*_*ijq*_)′ be a covariate vector of the *i*th subject (*i* = 1,…, *n*) observed at time *j* (*j* = 1,…, *t*), where *x*_*ijk*_ represents the *k*th covariate (*k* = 1,…, *q*) for given *i* and *j*. When the outcome is continuous, the use of MRM for this kind of data is common. A basic characteristic of these models is the inclusion of random subject effects into regression models in order to take into account the effect of subject on their repeated observations. These random subject effects, thus, describe each subject's trend across time and explain the correlational structure of longitudinal data. The model is written as

(1) $$\begin{array}{c} y_{ij}=\beta_{0j}+\beta_{1j}{\text{time}}_{ij}+\beta_{1}x_{ij1}+\cdots +\beta_{q}x_{ijq}+\varepsilon_{ij}, \end{array}$$

where some coefficients including *β*_0*j*_ and *β*_1*j*_ may be random [20].

 As we mentioned earlier, while MRM can handle the dropout, data must be complete within the given time point (in terms of both the dependent variable and covariates) for that time point to be included in the analysis. However, there are three naive approaches for handling missing in covariate which enables one to fit MRM models that assume that covariates are completely observed. These naive approaches are baseline measure throughout, carrying forward the last observation, and list-wise deletion (complete case analysis).

 In list-wise deletion method, cases with missing data were simply deleted from the data sets. This is the easiest approach in handling the missing covariates. In the other simple naive approach, baseline approach or baseline observation carried forward (BOCF) unobserved time-dependent covariates were replaced by baseline measure of those covariates. In this method, we assume that the time-dependent covariates have not changed since baseline. And finally, LOCF approach which simply fills the missing covariates data by carrying forward the last observed value and assuming that the values at the time of dropout are the same as those of the previous values. The question of interest is how much bias would arise by doing so. 

### 2.2. Seemingly Unrelated Regression Model

 Model (1) can be presented through a system of related equations by considering a set of *t* linear equations for each *t* time points. For simplicity, we assumed complete observation of outcome variables at all *t* time points.

 For expression of the model at each time point, let *y*_*j*_ be an *n* × 1 observed response vector and *X*_*jk*_ = (*x*_1*jk*_,…, *x*_*njk*_) be an *n* × *q* known vector at time *j*. If we assume that we observe the same number of variables for each time point, then the model in the population is defined as

(2) $$\begin{array}{c} y_{i1}=\alpha_{01}+\alpha_{1}x_{i11}+\cdots +\alpha_{q}x_{i1q}+\varepsilon_{i1},\\ y_{i2}=\alpha_{02}+\alpha_{1}x_{i21}+\cdots +\alpha_{q}x_{i2q}+\varepsilon_{i2},\\ \vdots \\ y_{it}=\alpha_{0t}+\alpha_{1}x_{it1}+\cdots +\alpha_{q}x_{itq}+\varepsilon_{it}. \end{array}$$

Or, generally

(3) yij=α0j+α1xij1+⋯+αqxijq+εij,j=1,2,…,t,  i=1,2,…,n.

This model (2) or (3) is based on Zellner's seemingly unrelated regression model. We devised a specific model for each time point, and this formulation introduced a system of correlated equations with different covariates at each observation time, some of which simultaneously could have time-invariant predictor variables such as sex. Nevertheless, correlation across the errors in different equations can provide links that can be exploited in estimation. 

 As presented in models (2) or (3), some restrictions were placed on the coefficients for each *k* covariate to be the same for all time points. By considering these restrictions, we can have a unique effect for each covariate as in MRM. The assumptions that we make about *ε*_*j*_ is that it is uncorrelated with the explanatory variables in all equations. In other words, we assumed that *ε*_*j*_ and *x*_*j*_ are orthogonal in the conditional mean sense [21].

 In model (2), we assumed that the same set of variables is observed for each time point which is usually an unreal assumption especially in medical studies because of intermittent observation of time-dependent covariates. Thus, some alternative approaches to handle sporadic or intermittent covariates are introduced.

 Consider that there are *n* subjects and *t* time points for which data on 3 variables including continuous outcome variable *Y*, a continuous time-dependent covariate *X*, and a time-invariant covariate *Z* were collected. For simplicity, consider that an outcome variable is measured completely at all *t* time points but the time-dependent covariate *X* is not measured, for example, at time *s*. We also assumed complete data on variable *Z*. Our proposed methods for this situation based on model (2) are presented in three models as follows. The easiest form is

(4) $$\begin{array}{c} y_{i1}=\alpha_{01}+\alpha_{1}z_{i}+\alpha_{2}x_{i1}+\varepsilon_{i1},\;j=1,\\ \vdots \\ y_{is}=\alpha_{0s}+\alpha_{1}z_{i}+\varepsilon_{is},\;j=s,\\ \vdots \\ y_{it}=\alpha_{0t}+\alpha_{1}z_{i}+\alpha_{2}x_{it}+\varepsilon_{it},\;j=t, \end{array}$$

where *i* represents the subject and *j* represents the time index. This is similar to complete case analysis of MRM, in which only the observed data without any imputations are used. However, there is a substantial difference; SUR model (4) uses all the available data, but MRM in complete case analysis deletes the observed *y*_*is*_ when the value of the covariate at that time point is missing.

 Secondly, the application of BOCF to SUR can be exemplarily described as

(5) $$\begin{array}{c} y_{i1}=\alpha_{01}+\alpha_{1}z_{i}+\alpha_{2}x_{i1}+\varepsilon_{i1},\;j=1,\\ \vdots \\ y_{is}=\alpha_{0s}+\alpha_{1}z_{i}+\alpha_{2}x_{i1}+\varepsilon_{is},\;j=s,\\ \vdots \\ y_{it}=\alpha_{0t}+\alpha_{1}z_{i}+\alpha_{2}x_{it}+\varepsilon_{it},\;j=t. \end{array}$$

Its difference from model (4) is in placing *x*_*i*1_ instead of unobserved covariate *x*_*is*_.

 And finally, the third approach is

(6) $$\begin{array}{c} y_{i1}=\alpha_{01}+\alpha_{1}z_{i}+\alpha_{2}x_{i1}+\varepsilon_{i1},\;j=1,\\ \vdots \\ y_{is}=\alpha_{0s}+\alpha_{1}z_{i}+\alpha_{2}x_{i(s-1)}+\varepsilon_{is},\;j=s,\\ \vdots \\ y_{it}=\alpha_{0t}+\alpha_{1}z_{i}+\alpha_{2}x_{it}+\varepsilon_{it},\;j=t, \end{array}$$

which is interpreted as the application of LOCF to SUR model. The difference between models (5) and (6) is the difference between *x*_*i*1_ and *x*_*i*(*s*−1)_ as the input in the right-hand side of *y*_*is*_.

 Similar to model (2), we assumed in all models (4), (5), and (6) that *ε*_*j*_ is uncorrelated with the explanatory variables in all equations (i.e., *E*(*ε*_*j*_ | *x*_*j*_) = 0, for *j* = 1,…, *t*) [21]. We considered the same coefficients for effect of *z* and *x* at all equations as *α*_1_ and *α*_2_, respectively. Furthermore, the parameter *α*_0*j*_ simply denotes a different aggregate time effect in each time point.

 Since the properties of SUR model in the presence of intermittently observed time-dependent covariates had not been evaluated in the previous studies, we investigated the behaviour of this model under various conditions by means of simulation studies and also compared its performance with MRM under three classic imputation methods, including complete case analysis, BOCF, and LOCF.

### 2.3. Simulation Study

 We studied the performances of the previously mentioned models, in terms of the amount of bias and MSE of the estimators, by simulation in SAS statistical package V 9.1 (SAS Inst., Cary, USA). The bias is the deviation in an estimate from the true quantity, which can indicate the performance of the methods being assessed, and the MSE provides a useful measure of the overall accuracy, as it incorporates both measures of bias and variability [22]. 

For simulation design, we used data from a psychiatric study by Hedeker et al. [23] which is extensively used in many methodological studies to examine the validity of the proposed models [24]. The objective of the study was to describe the longitudinal relationship between imipramine (IMI) and desipramine (DMI) plasma levels and clinical response in 66 depressed inpatients. Following a baseline placebo period of 1 week, patients received 225 mg/day doses of IMI for 4 weeks of the study. Additionally, the patients were rated with the Hamilton Depression Rating Scale (HDRS) [23] twice during the baseline placebo week (at the start, and end of this week) as well as at the end of each of the four treatment weeks of the study. These HDRS scores represent the dependent variables measured across time. Higher scores on HDRS represent higher levels of depression. Blood samples, drawn weekly for 4 weeks, 15 hours after the last drug intake of each patient, were assayed for IMI and DMI concentrations; these will be treated as time-dependent covariates.

 The sex and baseline depression were recorded for each patients. Diagnosis of the baseline depression classified as two types. The first type, nonendogenous or reactive depression, is associated with some tragic life events such as bereavement, whereas the second type, endogenous depression, is not a result of any specific event and appears to occur spontaneously. 

 A log transformation is used for IMI and DMI, since an inspection of the data indicated that the magnitude of these measurements varied greatly between individuals (from 4 to 312 mg/L for IMI and from 1 to 740 mg/L for DMI).

 Although the total number of subjects in this study was 66, we only considered 52 subjects that had all the measures at the end of four weeks, and it was coded as 0, 1, 2, and 3 for these respective time points. Because the purpose of the present study is to examine the performance of the proposed models for intermittently observed time-dependent covariates, other kinds of missing data such as attrition or missing in response variable were omitted. We applied different mixed-effect regression model for these data, but the best model (it has the smallest Akaike information criterion (AIC)) was random coefficient model.

 We generated complete data with different sample sizes (*n* = 20, 60, 120, and 240) from the random coefficient model

(7) HDRSij=(β0+u0)+(β1+u1)∗week +β2∗sex+β3∗Diag+β4∗IMIij +β5∗DMIij+εij.

Here *u*_0_ and *u*_1_ are subject-specific random effects for intercept and slope, respectively, which are from a normal distribution with a mean of 0 and variance of *σ*_0_^2^ and *σ*_1_^2^, respectively. *ε*_*ij*_ is the random error from a normal distribution with a mean of 0 and a variance of *σ* ^2^.

 The regression parameters *β*_0_, *β*_1_, *β*_2_, *β*_3_, *β*_4_, and *β*_5_ are the fixed effects for intercept, week, sex, Diag, IMI, and DMI, respectively. The estimated parameters of model (7) based on restricted maximum likelihood estimation (REML) method for 52 subjects are obtained as

(8) $$\begin{array}{c} \left(\begin{array}{c} {\hat{\beta }}_{0}\\ {\hat{\beta }}_{1}\\ {\hat{\beta }}_{2}\\ {\hat{\beta }}_{3}\\ {\hat{\beta }}_{4}\\ {\hat{\beta }}_{5} \end{array}\right)=\left(\begin{array}{c} 0.83\\ -1.97\\ -1.13\\ 0.23\\ -0.01\\ -1.12 \end{array}\right),\;\;\text{var}\left(\begin{array}{c} u_{0}\\ u_{1} \end{array}\right)=\left(\begin{array}{cc} 20.63 & 0.48\\ 0.48 & 2.59 \end{array}\right),\\ \varepsilon_{ij}\sim N\left(0,10.10\right), \end{array}$$

respectively. For this simulation study, we assumed both a continuous response and continuous time-dependent covariates. In order to simulate the trajectories of HDRS_*ij*_, it was necessary to simulate the trajectories of longitudinal covariates IMI_*ij*_ and DMI_*ij*_. We generated two time-dependent covariates of interest (IMI_*ij*_ and DMI_*ij*_) from linear regression against week with a random intercept,

(9) $$\begin{array}{c} {\text{IMI}}_{ij}=\left(\gamma_{01}+r_{01}\right)+\gamma_{11}\text{week}+\varepsilon_{ij1},\\ {\text{DMI}}_{ij}=\left(\gamma_{02}+r_{02}\right)+\gamma_{12}\text{week}+\varepsilon_{ij2}, \end{array}$$

where *γ*_01_, *γ*_02_, *γ*_11_, and *γ*_12_ are the fixed intercepts and slopes, respectively; *r*_01_, *r*_02_, *ε*_*ij*1_, and *ε*_*ij*2_ are from normal distribution with a mean of 0 and a variance of *δ*_01_, *δ*_02_, *δ*_11_ and *δ*_12_, respectively. We set

(10) $$\begin{array}{c} \left(\begin{array}{c} {\hat{\gamma }}_{01}\\ {\hat{\gamma }}_{11} \end{array}\right)=\left(\begin{array}{c} 3.39\\ 0.02 \end{array}\right),\;\;r_{01}\sim N\left(0,0.43\right),\\ \varepsilon_{ij1}\sim N\left(0,0.04\right),\\ \left(\begin{array}{c} {\hat{\gamma }}_{02}\\ {\hat{\gamma }}_{12} \end{array}\right)=\left(\begin{array}{c} 4.62\\ 0.07 \end{array}\right),\;\;r_{02}\sim N\left(0,0.57\right),\\ \varepsilon_{ij2}\sim N\left(0,0.04\right), \end{array}$$

for generating trajectories of IMI and DMI (based on REML estimated parameters of models (9)). Using these estimates and estimates from model (7), 500 simulations are carried out to generate complete data with different sample sizes. 

 The intermittent observations of time-dependent covariates are determined as follow. For simplicity, we assumed that all the subjects had complete data for their response variables and also for their time-dependent covariates at baseline and at the end. However, our interested time-dependent covariates, IMI_*ij*_ and DMI_*ij*_, might not be measured at weeks 1 or 2 or both time points for each covariate. By considering these kinds of missing data points and *t* = 4, we had 15 different scenarios for intermittent observation of time-dependent covariates.  Table 1 presents these scenarios.

 We based all the simulations reported on 500 replications as before. Each simulated data set with intermittently observed covariates was analyzed using different methods including MRM with complete case analysis, baseline value throughout, and last observation carried forward and also SUR models (4), (5), and (6). We indicated these methods by MRM_CC_, MRM_BOCF_, MRM_LOCF_, SUR_CC_, SUR_BOCF_, and SUR_LOCF_, respectively. Additionally, the performances of these methods were compared for all scenarios of intermittent observation of time-dependent covariates IMI and DMI. Moreover, we compared the performance of the proposed model with MRM for complete simulated data (simulated data sets with completely observation of time-dependent covariates and response variable).

The relative bias and mean square error of both the proposed models and MRM were computed in each step by $\overset{-}{\hat{\beta }}-\beta$ and$\;{\left(\overset{-}{\hat{\beta }}-\beta \right)}^{2}+{\left(\text{SE}(\hat{\beta })\right)}^{2}$, respectively. The estimation methods for the mixed model and the proposed SUR model were restricted maximum likelihood estimation (REML) and iterated seemingly unrelated regression (ITSUR), respectively. The ITSUR method uses information about contemporaneous correlation among error terms across equations in an attempt to improve the efficiency of parameter estimates [25]. It is drawn from the iteration of SUR method of estimation by recomputing the estimate of the cross-equation covariance matrix from the SUR residuals and then computing new SUR estimates based on this updated covariance matrix estimate. Continuing this iteration until convergence produces ITSUR estimates.

## 3. Results 

Tables 2, 3, 4, and 5 show the results from the simulation study. Table 2 presents the findings based on complete observation of time-dependent covariate, and Tables 3 to 5 show the findings based on three situations of intermittent observation of IMI and DMI from Table 1. The mean of estimated coefficients of interest, the amount of bias, and the mean square errors of these estimated coefficients based on different sample sizes of 20, 60, 120, and 240 quantify these findings. In addition, we calculate and report the naive approaches of handling intermittently observed time-dependent covariates IMI and DMI in Tables 3 to 5. The results from other situations of Table 1 were similar (not shown).

 In general, when the data are complete, the estimated parameters through the proposed model are similar to those of MRM for small, medium, and large sample sizes; also, it has little bias (less than 0.13) and MSE for all estimators of interest. Moreover, the differences between the two models become negligible as the sample size increases. The amount of bias of the proposed model for large sample sizes (120 or 240) is less than 0.04.

Table 3 presents the findings of MRM and the SUR model for the situation 1 for which we have complete observed values for HDRS and IMI, but DMI was measured only at baseline (0), 1, and 3 weeks.

Table 4 presents the coefficients, bias, and MSE of the estimators from both the proposed model and MRM for the situation 4. All values for HDRS and DMI were available for this situation, but IMI was measured only at baseline (0), 2 and 3 weeks.

Table 5 presents the performance of the proposed model compared to naive approaches of handling intermittent observed time-dependent covariates IMI and DMI for situation 9 for which we have measured HDRS at all weeks; however, DMI and IMI were measured only at baseline (0) and 3 weeks.

 Generally, the results for the parameters of interest are almost the same when we fit MRM and SUR models with BOCF and LOCF imputation methods, respectively. However, the best performance of these models in the simulated data for all sample sizes was fitted models based on LOCF imputation method. 

 The proposed BOCF and LOCF based on SUR model yielded nearly unbiased estimates of parameters (the amount of bias less than 0.15 in all cases). The LOCF approach in SUR model outperforms CC in every instance. However, the performance of LOCF and BOCF based methods for the SUR model was similar because, in our simulated data, there is a linear relationship between IMI and DMI through the weeks of the study.

 As shown in Tables 3, 4, and 5, the proposed SUR model by last observed measure of the covariate carried forward in the model yielded parameters with low bias even in situation 9 for which we observed IMI and DMI only at two time points. The amount of bias for SUR model based on LOCF approach was 0.07 or less for effects of IMI and DMI. Not surprisingly, parameters from the baseline approach are nearly as unbiased as the LOCF approaches; the amount of bias is less than 0.13.

 In MRM model, three naive approaches were used, and their performances were compared to each other and also to the proposed SUR model. MRM based on the complete case analysis led to bias of less than 0.2 for estimated parameters in each scenario. Complete case analysis based on SUR model in some of the situations performed unstable for parameters, with bias ranging from 0.01 to 0.93. This can occur because when we ignored some independent variables in the regression models, it decreased the determination coefficient of the model. However, the amount of bias that is considered troublesome has varied from $\left(1/2\right)\text{SE}(\hat{\beta })$ [26] to $2\text{SE}(\hat{\beta })$ [27] in different studies. The amounts of bias for most of the estimated coefficients in the present study were even smaller than $\left(1/2\right)\text{SE}(\hat{\beta })$. 

 The MRM based on LOCF approach performs as well as SUR model based on LOCF approach, with amount of bias less than 0.16. The baseline value carried forward in MRM performed similar to LOCF approach. The amount of bias was not substantially decreased if we increased the sample size from 20 to 240.

 In general, when the bias is nearly the same, the MSEs of the estimated coefficients in SUR model are slightly larger than those in MRM. However, this difference becomes smaller and smaller as the sample size increases. 

## 4. Worked Example

 This example is motivated by our research on evaluating the changes of biochemical levels of magnesium (Mg), blood urea nitrogen (BUN), and full history and clinical assessment of serum creatinine (Cr), albumin, hemoglobin, calcium (Ca), sodium (Na), potassium (K), and phosphorous (P) and their relation to intradialytic hypotension (IDH) in 21 chronic hemodialysis patients in a midweek single dialysis session. According to national kidney foundation guideline (NFK-DOQI guideline), IDH was defined as a decrease of systolic blood pressure of more than 20 mmHg of the basal value [27, 28]. Systolic blood pressure and serial assessment of magnesium, potassium, phosphorus, calcium, and sodium were measured at the start of the hemodialysis session, 2 hours later, and at the end of the session. Among biochemical determining factors, the levels of BUN and weight were measured at the start and the end of the session and clinical examination of the other factors such as serum creatinine, albumin, and haemoglobin were measured only at the start of the session. This fact motivated us to examine the performance of the mentioned methods of the present study in this data set.

 In the first step of the analysis, covariates that have *P* value under 0.25 in the univariate analysis (the results were not shown) were considered in the last model which are levels of Cr, K, P, BUN, and weight. The intermittently observed time-dependent covariate of interest in this example is blood urea nitrogen and weight. Based on AIC index, the best fitted model is mixed-effect regression model with random effect of intercept. 

Table 6 presents the estimated coefficients and standard error (SE) of each covariate from different proposed models. As seen in the table, there is similarity between both estimated coefficients and their standard errors from the proposed model as compared with the mixed-effect regression model in spite of small sample size (*n* = 21). These findings are consistent with the results from our simulation study. Consider the point that SUR model based on LOCF and BOCF leads to the same result due to the fact that for unobserved value of BUN after 2 hours the last observation and baseline observation being the same. This situation also happens for MRM. Thus, we only present the findings for BOCF method in Table 6.

 Additionally, among considered variables in the last model, the effect of blood urea nitrogen is significant through all models.

## 5. Discussion 

 Intermittently observed time-dependent covariates are relatively common situation in practice. Sometimes, these covariates are measured intermittently for all of the subjects. This situation happens in medical applications commonly. This specific research question about analyzing longitudinal data with a special kind of intermittently observed time-dependent covariate was our motivation for the present study. We proposed a general class of multivariate linear model with respect to each observation time. The proposed model is based on SUR model, and it introduces several correlated multivariate linear models with some similar and different covariates at each observation time, like the model proposed by Park and Woolson [19]. However, they considered that if *y*_*ij*_ is observed, then *x*_*ij*_ is always observed, and missing observations are assumed to be missing at random. We considered some restrictions on the parameters to be the same for all similar covariates at each observation time, and it led to fewer parameters and obtaining a unique estimate for each covariate as mixed model. Furthermore, the advantages of this model are its simple applicability, faster speed of convergence, and the ease of estimation. The other desirable feature of this method is that it does not require any knowledge about the covariance structure and the distribution of random effects unlike mixed-effect regression model. The presented models are quite standard and are not novel, but we attempt to expose these models to medical applications. Up to our knowledge, there is no comprehensive approach for such intermittent observation of time-dependent covariates in medical studies based on the proposed SUR models.

 Besides the main objectives of the study to handle intermittent observation of time-dependent covariates in longitudinal data, we examined the performance of SUR based model for longitudinal data with covariates measured at the same time points. For complete data, our simulation study showed that the SUR model performs as efficient as the MRM even in a small sample size. Moreover, SUR models converge faster than MRM. These simulation results are consistent with the models of Patel [29] and Verbyla and Venables for complete and balanced longitudinal data [30]. However, they considered other estimation methods, and also they did not compare models to MRM. 

 Park and Woolson proposed a SUR model for handling incomplete and unbalanced longitudinal data [19]. They considered two methods of estimation: one method is the generalized least square (GLS) and the other iterative maximum likelihood estimation (MLE) using the EM algorithm. In this study, we used ITSUR method as our estimation method, and other kinds of classic imputation methods such as BOCF or LOCF. In SUR model when we have both different variables and some similar ones simultaneously for different response variables, ITSUR is one of the best methods of estimation. Moreover, the two main motivations for the use of ITSUR as our estimation method are (1) the use of information about contemporaneous correlation among error terms across equations in an attempt to improve the efficiency of parameter estimates, and (2) imposition of restrictions in the involved parameters in different equations. 

 In the case of longitudinal data with intermittent observation of time-dependent covariates, at each time points, we considered a univariate model for each measured response and also the observed measure of each time-dependent covariate as an independent variable at that time point. For unobserved value of the time-dependent covariate at that specific time point, we considered 3 easy methods. Similar to SUR models for complete observed time-dependent covariate, we imposed restriction on the parameters to be the same at all time points for that covariate. We put this restriction on all the time-invariant covariates such as sex as well. Our findings demonstrate that sometimes some bias can arise from fitting the SUR model. However, based on the study of Sinharay et al., this amount of bias could be negligible because in all situations it is smaller than $2\text{SE}(\hat{\beta })$.

 Among the introduced model for intermittent covariates in longitudinal data, SUR model with last observation carried forward has the best desirable performance. Moreover, through this method, in all situations of Table 1, the amount of bias was less than $\left(1/2\right)\text{SE}(\hat{\beta })$. These findings are consistent with MRM with LOCF method.

 There many situations in medical and behavioral research in which variables of interest are measured intermittently. Oftentimes, these kinds of data are analyzed through MRM. However, it is common place for educational researchers to conduct SUR model for analyzing these kinds of datasets. SUR models are underutilized and should be given more consideration as an analytic technique due to their elegant features. 

 There are some further points which are worth noting to mention here. Firstly, in generating time-dependent covariates, we considered the linear relationship between variable week and time-dependent covariates of interest (IMI and DMI) based on Reisby's dataset. Even so, sometimes the time-dependent covariates change through the time by curve linear relation. These kinds of relationships should be a consideration of future investigations. Secondly, we restricted our study to only continuous response and time-dependent covariates. The application of the proposed method for categorical responses or covariates could be investigated in future studies.

Thirdly, we considered three classic imputation approaches, and performances of other kinds of imputation methods such as multiple imputations could be studied in the forthcoming investigations. Finally, we simply assumed complete observation of the response variable, and we did not address other kinds of missing covariates such as attrition. Thus, future studies could examine the performance of the proposed models in the presence of other kinds of missing covariate in addition to intermittent observation of the covariates in more depth. 

## 6. Conclusion

 The SUR model performs useful analysis of longitudinal data with completely observed time-dependent covariates as MRM. This model also shows desirable performance in presence of intermittently observed time-dependent covariates even for small to medium sample sizes. Moreover, the desirable features of these models including simplicity, faster speed of convergence, and no need for knowledge about the covariance structure and the distribution of random effects unlike mixed-effect regression model make these models applicable in experimental and medical studies. 

## Figures and Tables

**Table 1:** Specification of all situations of intermittently observed time-dependent covariates to be imposed on IMI and DMI.

Situation	Covariate							
	IMI				DMI			
	Week							
	0	1	2	3	0	1	2	3
1	O	O	O	O	O	O	U	O
2	O	O	U	O	O	O	O	O
3	O	O	O	O	O	U	O	O
4	O	U	O	O	O	O	O	O
5	O	O	O	O	O	U	U	O
6	O	U	U	O	O	O	O	O
7	O	U	O	O	O	U	O	O
8	O	O	U	O	O	O	U	O
9	O	U	U	O	O	U	U	O
10	O	O	U	O	O	U	O	O
11	O	U	O	O	O	O	U	O
12	O	U	U	O	O	U	O	O
13	O	U	U	O	O	O	U	O
14	O	U	O	O	O	U	U	O
15	O	O	U	O	O	U	U	O

*Note: “O” means that the covariate at that time point was “Observed”, and “U” means that it was “Unobserved” at that time point.

**Table 2:** Simulation results (summary of estimated parameters, bias, and MSE) for complete observation of time-dependent covariates with different sample sizes (*n* = 20,60,120, and 240).

Parameter (true value)	Method	Sample size = 20			Sample size = 60			Sample size = 120			Sample size = 240		
		$\overset{↼}{\beta }$	Bias	MSE	$\overset{↼}{\beta }$	Bias	MSE	$\overset{↼}{\beta }$	Bias	MSE	$\overset{↼}{\beta }$	Bias	MSE
*β*_2_	REML	−1.01	.12	7.24	−1.14	−.009	1.89	−1.12	.01	.828	−1.14	−.006	.42
(−1.13)	ITSUR	−1.003	.13	7.37	−1.14	−.009	1.89	−1.12	.01	.829	−1.14	−.006	.42

*β*_3_	REML	.18	−.04	7.33	.14	−.09	1.90	.21	−.02	.864	.25	.02	.42
(.23)	ITSUR	.18	−.05	7.40	.14	−.09	1.89	.21	−.02	.863	.25	.02	.42

*β*_4_	REML	.03	.04	3.32	−.01	−.004	.82	−.02	−.009	.39	−.02	−.009	.18
(−.01)	ITSUR	.02	.03	3.39	−.009	.0004	.83	−.02	−.008	.39	−.02	−.009	.18

*β*_5_	REML	−1.11	.006	2.25	−1.12	−.003	.57	−1.08	.04	.26	−1.10	.02	.13
(−1.12)	ITSUR	−1.11	.01	2.36	−1.12	−.005	.58	−1.09	.03	.27	−1.10	.02	.13

*Note: REML represents the estimated parameters from MRM, and ITSUR represents that of the SUR model.

**Table 3:** Simulation results (summary of estimated parameters, bias, and MSE) for intermittent observation of time-dependent covariates (situation 1 from Table 1) with different sample sizes (*n* = 20,60,120, and 240).

Parameter (true value)	Method	Sample size = 20			Sample size = 60			Sample size = 120			Sample size = 240		
		$\overset{↼}{\beta }$	Bias	MSE	$\overset{↼}{\beta }$	Bias	MSE	$\overset{↼}{\beta }$	Bias	MSE	$\overset{↼}{\beta }$	Bias	MSE
*β*_2_ (−1.13)	SUR_CC_	−.94	.19	7.25	−1.15	−.02	1.87	−1.12	.008	.83	−1.13	−.006	.43
	MRM_CC_	−1.003	.13	6.39	−1.14	−.01	1.86	−1.12	.009	.84	−1.14	−.01	.43
	SUR_BOCF_	−.99	.13	7.26	−1.14	−.01	1.89	−1.12	.01	.83	−1.14	−.007	.42
	MRM_BOCF_	−1.01	.12	6.05	−1.16	−.03	1.83	−1.12	.007	.82	−1.14	−.009	.42
	SUR_LOCF_	−.98	.14	7.40	−1.14	−.01	1.88	−1.12	.01	.83	−1.14	−.007	.42
	MRM_LOCF_	−1.01	.12	6.18	−1.16	−.03	1.82	−1.12	.009	.83	−1.14	−.01	.42

*β*_3_ (.23)	SUR_CC_	.20	−.03	7.22	.15	−.08	1.90	.20	−.03	.87	.24	.009	.43
	MRM_CC_	.17	−.06	6.08	.14	−.09	1.90	.20	−.03	.87	.24	.01	.43
	SUR_BOCF_	.21	−.02	7.29	.14	−.09	1.90	.21	−.02	.86	.24	.01	.42
	MRM_BOCF_	.12	−.11	5.88	.12	−.11	1.83	.20	−.03	.85	.24	.01	.42
	SUR_LOCF_	.21	−.02	7.37	.12	−.10	1.85	.21	−.02	.85	.24	.01	.42
	MRM_LOCF_	.13	−.1	5.91	.12	−.11	1.82	.19	−.04	.85	.24	.01	.42

*β*_4_ (−.01)	SUR_CC_	.002	.01	3.42	−.02	−.006	.82	−.02	−.01	.39	−.02	.39	.18
	MRM_CC_	−.02	−.01	3.24	−.05	−.04	.91	−.03	−.02	.43	−.02	−.009	.18
	SUR_BOCF_	.03	.04	3.39	−.01	−.001	.83	−.02	−.01	.39	−.02	−.01	.18
	MRM_BOCF_	−.03	−.02	2.67	−.02	−.007	.77	−.02	−.01	.37	−.02	−.01	.17
	SUR_LOCF_	.02	.02	3.39	−.009	.0004	.83	−.02	−.008	.39	−.02	−.01	.18
	MRM_LOCF_	−.03	−.02	2.68	−.02	−.0006	.76	−.02	−.01	.37	−.02	−.01	.17

*β*_5_ (−1.12)	SUR_CC_	−.45	.67	1.56	−.49	.63	.66	−.45	.67	.58	−.46	.66	.51
	MRM_CC_	−1.16	−.04	2.20	−1.13	−.009	.61	−1.08	.04	.28	−1.10	.02	.14
	SUR_BOCF_	−1.03	.09	2.37	−1.03	.09	.60	−.99	.13	.29	−1.02	.10	.14
	MRM_BOCF_	−.97	.15	1.81	−.98	.14	.55	−.95	.17	.29	−.97	.14	.14
	SUR_LOCF_	−1.06	.06	2.48	−1.05	−.07	.58	−1.006	.11	.27	−1.02	.09	.14
	MRM_LOCF_	−1.06	.06	1.96	−1.03	−.09	.54	−.99	.13	.27	−1.01	.10	.14

*Note: SUR_CC_, MRM_CC_, SUR_BOCF_, MRM_BOCF_, SUR_LOCF_,  and MRM_LOCF_ represent the estimated parameters from MRM and SUR models under complete case (CC) analysis, baseline observation carried forward (BOCF), and last observation carried forward (LOCF) method, respectively.

**Table 4:** Simulation results (summary of estimated parameters, bias, and MSE) for intermittent observation of time-dependent covariates (situation 4 from Table 1) with different sample sizes (*n* = 20,60,120, and 240).

Parameter (True value)	Method	Sample size = 20			Sample size = 60			Sample size = 120			Sample size = 240		
		$\overset{↼}{\beta }$	Bias	MSE	$\overset{↼}{\beta }$	Bias	MSE	$\overset{↼}{\beta }$	Bias	MSE	$\overset{↼}{\beta }$	Bias	MSE
*β*_2_ (−1.13)	SUR_CC_	−1.002	.13	7.13	−1.14	−.01	1.87	−1.12	.01	.83	−1.13	−.005	.42
	MRM_CC_	−1.01	.13	6.75	−1.17	−.04	1.94	−1.13	.002	.87	−1.14	−.007	.44
	SUR_BOCF_	−1.02	.11	7.42	−1.14	−.01	1.88	−1.12	.01	.83	−1.14	−.006	.42
	MRM_BOCF_	−1.03	.10	6.22	−1.16	−.03	1.82	−1.12	.01	.83	−1.14	−.009	.42
	SUR_LOCF_	−1.02	.11	7.42	−1.14	−.01	1.88	−1.12	.01	.83	−1.14	−.006	.42
	MRM_LOCF_	−1.03	.10	6.22	−1.16	−.03	1.82	−1.12	.01	.83	−1.14	−.009	.42

*β*_3_ (.23)	SUR_CC_	.18	−.05	7.17	.14	−.09	1.88	.21	−.02	.86	.24	.01	.43
	MRM_CC_	.16	−.07	6.32	.12	−.11	1.94	.19	−.03	.89	.23	.004	.44
	SUR_BOCF_	.18	−.05	7.59	.14	−.08	1.89	.21	−.02	.86	.25	.01	.42
	MRM_BOCF_	.15	−.08	5.97	.13	−.10	1.81	.20	−.03	.85	.24	.01	.42
	SUR_LOCF_	.18	−.05	7.59	.14	−.08	1.89	.21	−.02	.86	.25	.01	.42
	MRM_LOCF_	.15	−.08	5.97	.13	−.10	1.81	.20	−.03	.85	.24	.01	.42

*β*_4_ (−.01)	SUR_CC_	−.08	−.07	1.79	−.02	−.009	.47	−.02	−.01	.23	−.01	−.004	.10
	MRM_CC_	−.02	−.01	3.36	.01	.02	.93	−.01	−.004	.45	−.02	−.008	.19
	SUR_BOCF_	.04	.05	3.62	−.02	.02	.81	−.01	−.0004	.41	−.02	−.008	.18
	MRM_BOCF_	−.02	−.01	2.76	.009	.02	.76	−.01	−.004	.39	−.02	−.008	.17
	SUR_LOCF_	.04	.05	3.62	−.02	.02	.81	−.01	−.0004	.41	−.02	−.008	.18
	MRM_LOCF_	−.02	−.01	2.76	.009	.02	.76	−.01	−.004	.39	−.02	−.008	.17

*β*_5_ (−1.12)	SUR_CC_	−1.10	.02	2.27	−1.12	−.005	.57	−1.08	.03	.27	−1.10	.02	.13
	MRM_CC_	−1.13	−.01	2.40	−1.15	−.03	.64	−1.09	.03	.32	−1.10	.02	.15
	SUR_BOCF_	−1.11	.006	2.39	−1.12	−.004	.57	−1.08	.03	.27	−1.10	.02	.13
	MRM_BOCF_	−1.14	−.02	1.87	−1.13	−.006	.54	−1.08	.04	.26	−1.10	.02	.13
	SUR_LOCF_	−1.11	.006	2.39	−1.12	−.004	.57	−1.08	.03	.27	−1.10	.02	.13
	MRM_LOCF_	−1.14	−.02	1.87	−1.13	−.006	.54	−1.08	.04	.26	−1.10	.02	.13

*Note: SUR_CC_, MRM_CC_, SUR_BOCF_, MRM_BOCF_, SUR_LOCF_,  and MRM_LOCF_ represent the estimated parameters from MRM and SUR models under complete case (CC) analysis, baseline observation carried forward (BOCF), and last observation carried forward (LOCF) method, respectively.

**Table 5:** Simulation results (summary of estimated parameters, bias, and MSE) for intermittent observation of time-dependent covariates (situation 9 from Table 1) with different sample sizes (*n* = 20, 60,120, and 240).

Parameter (true value)	Method	Sample size = 20			Sample size = 60			Sample size = 120			Sample size = 240		
		$\overset{↼}{\beta }$	Bias	MSE	$\overset{↼}{\beta }$	Bias	MSE	$\overset{↼}{\beta }$	Bias	MSE	$\overset{↼}{\beta }$	Bias	MSE
*β*_2_ (−1.13)	SUR_CC_	−.95	.18	6.64	−1.14	−.01	1.86	−1.12	.01	.83	−1.13	−.003	.43
	MRM_CC_	−.99	.14	7.43	−1.16	−.03	2.007	−1.13	.002	.89	−1.14	−.01	.46
	SUR_BOCF_	−1.04	.09	7.90	−1.14	−.01	1.92	−1.12	.008	.83	−1.14	−.006	.42
	MRM_BOCF_	−1.03	.10	6.53	−1.16	−.03	1.85	−1.12	.006	.82	−1.14	−.01	.42
	SUR_LOCF_	−1.04	.09	7.90	−1.14	−.01	1.92	−1.12	.008	.83	−1.14	−.006	.42
	MRM_LOCF_	−1.03	.10	6.53	−1.16	−.03	1.85	−1.12	.006	.82	−1.14	−.01	.42

*β*_3_ (.23)	SUR_CC_	.20	−.03	6.77	.14	−.09	1.90	.20	−.02	.88	.24	.007	.44
	MRM_CC_	.20	−.02	6.70	.12	−.11	2.008	.19	−.04	.93	.23	−.002	.45
	SUR_BOCF_	.24	.01	7.53	.14	−.08	1.93	.21	−.02	.87	.24	.01	.42
	MRM_BOCF_	.17	−.05	5.95	.12	−.11	1.85	.19	−.03	.86	.24	.01	.43
	SUR_LOCF_	.24	.01	7.53	.14	−.08	1.93	.21	−.02	.87	.24	.01	.42
	MRM_LOCF_	.17	−.05	5.95	.12	−.11	1.85	.19	−.03	.86	.24	.01	.43

*β*_4_ (−.01)	SUR_CC_	−.11	−.10	1.58	−.007	.003	.41	−.02	−.01	.20	−.02	−.005	.09
	MRM_CC_	−.02	−.008	4.16	.01	.003	1.13	−.03	−.02	.56	−.02	−.02	.25
	SUR_BOCF_	.02	.03	4.10	.02	.02	.93	−.007	.002	.46	−.01	−.001	.20
	MRM_BOCF_	−.03	−.02	3.38	.02	.03	.88	.002	.01	.44	.005	.01	.20
	SUR_LOCF_	.02	.03	4.10	.02	.02	.93	−.007	.002	.46	−.01	−.001	.20
	MRM_LOCF_	−.03	−.02	3.38	.02	.03	.88	.002	.01	.44	.005	.01	.20

*β*_5_ (−1.12)	SUR_CC_	−.19	.93	2.02	−.28	.84	1.001	−.27	.85	.86	−.26	.86	.80
	MRM_CC_	−1.14	−.02	2.89	−1.16	−.04	.77	−1.09	.03	.37	−1.10	.02	.18
	SUR_BOCF_	−1.05	.07	2.82	−1.02	−.09	.71	−.96	.15	.34	−.99	.13	.17
	MRM_BOCF_	−.95	.16	2.27	−.97	.15	.65	−.92	.20	.35	−.94	.18	.18
	SUR_LOCF_	−1.05	.07	2.82	−1.02	−.09	.71	−.96	.15	.34	−.99	.13	.17
	MRM_LOCF_	−.95	.16	2.27	−.97	.15	.65	−.92	.20	.35	−.94	.18	.18

*Note: SUR_CC_, MRM_CC_, SUR_BOCF_, MRM_BOCF_, SUR_LOCF_,  and MRM_LOCF_ represent the estimated parameters from MRM and SUR models under complete case (CC) analysis, baseline observation carried forward (BOCF), and last observation carried forward (LOCF) method, respectively.

**Table 6:** Summary of the estimated parameters and standard errors of different models for risk factors of intradialytic hypotension among hemodialysis patients during a dialysis session.

Covariate	MRM_CC_	SUR_CC_	MRM_BOCF_	SUR_BOCF_
	coefficient (SE)	coefficient (SE)	coefficient (SE)	coefficient (SE)
Cr (mg/dL)	0.20 (0.18)	0.18 (0.18)	0.19 (0.18)	0.17 (0.17)
Weight (kg)	0.39 (0.26)	0.12 (0.16)	0.49 (0.25)	0.44 (0.24)
BUN (mg/dL)	−0.42 (0.15)	−0.31 (0.15)	−0.33 (0.10)	−0.29 (0.16)
K (mg/dL)	7.19 (6.66)	8.13 (6.19)	6.87 (5.33)	6.01 (6.05)
P (mg/dL)	1.23 (2.58)	−1.72 (1.70)	−0.66 (1.52)	−1.39 (1.72)

*Note: Cr: creatinine; BUN: blood urea nitrogen; K: potassium; P: phosphorus.

## Abbreviations

BUN: Blood urea nitrogen
BOCF: Baseline observation carried forward
CC: Complete case
DMI: Desipramine
HDRS: Hamilton Depression Rating Scale
IMI: Imipramine
ITSUR: Iterated seemingly unrelated regression
LOCF: Last observation carried forward
MRM: Mixed-effect regression model
MSE: Mean square error
REML: Restricted Maximum Likelihood
SE: Standard error
SUR: Seemingly unrelated regression.
